# Medical students’ personal choice for mode of delivery in Santa Catarina, Brazil: a cross-sectional, quantitative study

**DOI:** 10.1186/1472-6920-12-57

**Published:** 2012-07-20

**Authors:** Tatiane Watanabe, Roxana Knobel, Guilherme Suchard, Mario Julio Franco, Eleonora d’Orsi, Elenice Bertanha Consonni, Marcos Consonni

**Affiliations:** 1 Medical student, University of Santa Catarina (UFSC), Florianópolis, Brazil; 2 Department of Gynecology and Obstetrics, University of Santa Catarina (UFSC), Florianópolis, Brazil; 3 Departament of Public Health, University of Santa Catarina (UFSC), Florianópolis, Brazil; 4 Department of Neurology, Psychology and Psychiatry, Botucatu School of Medicine, Universidade Estadual Paulista (Unesp), Botucatu, Brazil; 5 Department of Gynecology and Obstetrics, Botucatu School of Medicine, Universidade Estadual Paulista (Unesp), Botucatu, Brazil

**Keywords:** Cesarean section, Women’s healthcare, Medical education, Obstetrics

## Abstract

**Background:**

The increase in overall rates of cesarean sections (CS) in Brazil causes concern and it appears that multiple factors are involved in this fact. In 2009, undergraduate students in the first and final years of medical school at the University of Santa Catarina answered questionnaires regarding their choice of mode of delivery. The aim of the study was to evaluate whether the education process affects decision-making regarding the waay of childbirth preferred by medical students.

**Methods:**

A cross-sectional, quantitative study was conducted based on data obtained from questionnaires applied to medical students. The questions addressed four different scenarios in childbirth, as follows: under an uneventful pregnancy; the mode of delivery for a pregnant woman under their care; the best choice as a healthcare manager and lastly, choosing the birth of their own child. For each circumstance, there was an open question to explain their choice.

**Results:**

A total of 189 students answered the questionnaires. For any uneventful pregnancy and for a pregnant woman under their care, 8.46% of the students would opt for CS. As a healthcare manager, only 2.64% of the students would recommend CS. For these three scenarios, the answers of the students in the first year did not differ from those given by students in the sixth year. In the case of the student’s own or a partner’s pregnancy, 41.4% of those in the sixth year and 16.8% of those in the first year would choose a CS. A positive association was found between being a sixth year student and a personal preference for CS according to logistic regression (OR = 2.91; 95%CI: 1.03–8.30). Pain associated with vaginal delivery was usually the reason for choosing a CS.

**Conclusions:**

A higher number of sixth year students preferred a CS for their own pregnancy (or their partner’s) compared to first year students. Pain associated with vaginal delivery was the most common reason given for haven chosen a CS. The students’ preference for childbirth changed over time during their graduation in favor of cesarean sections. This finding deserves considerable attention when structuring medical education in Obstetrics.

## Background

Cesarean sections undeniably constitute an important tool in ensuring the safety of the mother and fetus when complications develop. Nevertheless, analysis of the current Cesarean section rates in Brazil [1] causes concern. The increase in the rates does not appear to be due to an increase only in the clinical indications. There appear to be other factors involved in the preference for this mode of childbirth. This hypothesis is strengthened by the observation that Cesarean rates are higher in women with better schooling and greater family income, who, in principle, should represent a lower gestational risk [2-4].

Studies on the etiology of this phenomenon have reported that pregnant women, even those with no pathology, prefer surgical childbirth [5-7]. Fear of the pain associated with vaginal delivery, uncertainty with respect to her sexual life following delivery and the belief that this route of delivery is more unpredictable and therefore more risky for the infant are factors that are said to contribute to women’s preference for a Cesarean section [8-10].

In contradiction with this tendency to attribute the rise in the number of Cesarean sections to maternal demand, studies conducted within the last ten years show that Brazilian women do not prefer surgical delivery [11-16]. Bearing in mind that neither women’s preferences nor true clinical indications can justify such high rates of Cesarean section, it has to be considered that at least a proportion of healthy women who want to have their babies by vaginal delivery are undergoing surgery in Brazil. The physician’s role in this scenario must then be taken into consideration.

Studies have confirmed that it was the medical community that was largely responsible for the beginning of the Cesarean epidemic, more specifically the obstetricians, based on the technocratic model of healthcare [11,15,17]. According to the technocratic principle that aggressive and technological interventions are required to control nature, in itself unpredictable and dangerous, childbirth has been divided into stages, each of which must be submitted to medical control [18]. If the dilatation stage takes longer than usual, oxytocin is introduced. If there is any difficulty in the expulsion stage, episiotomy is performed. If, despite these interventions, delivery still fails to follow the standard stages, a Cesarean section is then performed. Physicians may opt for interventions and Cesarean sections not simply because these procedures provide them with better conditions for managing their other activities and consequently better remuneration, in addition to the belief that Cesarean sections are better and cause less suffering to the woman and infant [11,15,17], but also because of their own insecurity. As years go by, a loss of expertise and capability for dealing with vaginal deliveries and their possible complications may be occurring [17]. A fact that supports this hypothesis is the finding that in several countries preference for a Cesarean section is greater among physicians than in the general population. In 1997, in one of the pioneering studies conducted on this subject, Al-Mufti, McCarthy and Fish [19] reported that 17% of obstetricians participating in a study in London preferred a Cesarean section even when no complications were present. Interviews conducted in a hospital in Turkey in 2007 [20] found that more than half the healthcare professionals (physicians, nurses, midwives and laboratory technicians) believed that Cesarean sections were safer for the baby. In Norway, a study conducted in 2008 found that more than a quarter of obstetricians/gynecologists had already been submitted to a Cesarean section compared to a rate of only 12% in the general population [21].

If it is true that: *as long as so many physicians prefer to have their children by Cesarean section, rates are unlikely to fall*[21], an affirmation in which it is understood that the physician’s personal choice is reflected in his/her professional conduct when counseling a pregnant woman on childbirth, the reasons for the preference of these professionals for surgical delivery of their own children must be evaluated.

Historically, the teaching of medicine was based on forming specialists in various areas; however, for decades now the possibility has been discussed of preparing physicians for primary healthcare, as general practitioners, equipped to meet the demands of family and community medicine. At the end of the 1980s and the beginning of the 1990s, this process was intensified with the institution of Brazil’s National Health Service, the Unified Health System (SUS) [22]. The undergraduate medical course of the Federal University of Santa Catarina (UFSC) is currently undergoing a transition phase in which a medicalized and hospital-based environment is confronted by a prototype of medical education focused on primary healthcare. Selected together with other medical schools throughout Brazil, the UFSC School of Medicine has been receiving financial support since 2003 from a program known as PROMED aimed at promoting curricular changes in medical education [23] with the objective of creating a process of change focused on the health requirements of the population and the SUS. PROMED functions in compliance with the guidelines established for the national curriculum of undergraduate medical courses, published in November 2001. Article 3 of the act that instituted these curricular guidelines establishes the profile of the medical graduate as generalist, humanist, critical and reflexive, trained to take actions based on ethical principles within the health-disease process at different healthcare levels, with actions aimed at promoting, recovering and rehabilitating health and preventing disease within the perspectives of the integrality of healthcare, with a sense of social responsibility and commitment to society to promote the integral health of human beings [24].

At the time of data collection, the curriculum of the undergraduate medical course at the Federal University of Santa Catarina consisted of an obligatory 9,900 hours of classes, 5,760 of which referred to the two-year medical internship. Prior to beginning internship, students should complete 648 hours of classes on women’s healthcare, while during internship gynecology/obstetrics should correspond to 1,248 hours of classes [25]. The internships are conducted at the *Carmela Dutra* Maternity Hospital (3,569 deliveries and a Cesarean section rate of 44.3% in 2010) [1] and in the university teaching hospital (1,817 deliveries and a Cesarean section rate of 38% in 2010) [1].

In view of the need to reduce the rates of unnecessary Cesarean sections (Cesarean sections with no indication or clinical justification) both in Santa Catarina and in Brazil as a whole, it is essential to acquire information on the reasons that motivate physicians to carry out Cesarean sections rather than vaginal deliveries. Therefore, the objective of the present study was to evaluate whether the education process for undergraduate medical students at UFSC affects this decision-making. To do so, the opinion of two groups of students, one in the first year and the other in the sixth and final year of medical school at the Federal University of Santa Catarina in 2009, was compared with respect to their preferred mode of delivery.

## Methods

To compare the two groups, a cross-sectional, quantitative study was conducted based on data obtained from questionnaires applied to all students in the first and final year of the undergraduate medical course at the Federal University of Santa Catarina during the second semester of 2009.

The dependent variables that generated responses of “vaginal delivery” or “Cesarean section” were:

 a) What mode of delivery offers fewer risks and greater benefits in a normal pregnancy (when no pathology is present)?

 b) What mode of delivery would you recommend for a normal pregnant woman under your medical care (when no pathology is present)?

 c) What mode of delivery would you recommend for the general population (for healthy women with no pathology) if you were a healthcare manager?

 d) What mode of delivery would you choose for the birth of your own (or your partner’s) child?

For each dependent variable, there was an open question in which the respondent was requested to explain the reason for their choice.

The main independent variable that defined the two groups of students was being either in the first or the sixth undergraduate year at the beginning of the study.

Control variables consisted of: gender, age, monthly family income, the population of the student’s home town, [26] skin color, personal obstetric history and how the student was born (Cesarean/vaginal).

The data obtained were entered and stored in a database using the EpiData Entry software program, version 3.1. The data were analyzed using the SPSS statistical software package. To calculate the differences between the groups, the chi-square test, Student’s *t*-test and Fisher’s exact test were used as appropriate. Any unanswered variables in the questionnaires were excluded from the analysis.

To investigate whether some of the study variables affected the sixth-year students’ preference for that form of childbirth in the case of their own pregnancy or that of their partner, multiple binary logistic regression was performed, with estimated odds ratios (OR) and their respective 95% confidence intervals. The outcome variable was the preference for Cesarean section in the case of a student’s own pregnancy or that of his partner. The main exposure variable was the year of medical school and the control variables consisted of: gender, monthly family income, age group and skin color. The backward elimination was used.

The open responses were analyzed and categorized and the reasons given for the student’s preference for the mode of delivery of their own child are presented and discussed.

This study was conducted entirely in accordance with the guidelines outlined in Resolution 196/96 of the National Health Council. The protocol was submitted to and approved by the institution’s Internal Review Board under Process 260/09.

## Results

A total of 189 students completed a questionnaire, 97 students in the first year and 92 in the final year of medical school. Four first-year students and six final-year students did not participate in the study.

Based on the distribution of the control variables (Table 1), statistically significant differences were found between the two groups of students with respect to family income, skin color and gender. In addition, there was a significant difference in age between the groups that was expected: the mean age of the first-year students being 21.16 ± 3.07 years (mean ± standard deviation) compared to 25 ± 2.17 years for the sixth-year students (p < 0.001; Student’s *t*-test) (data not shown in tables).

**Table 1:** Distribution of the control variables according to the year of medical school

**Variable**	**First-Year**		**Sixth-Year**		p-value^*^
	**n**	**%**	**n**	**%**	
**Gender**					
Male	61	62.9	40	43.5	**0.008**
Female	36	37.1	52	56.5	
Monthly family income**					
< 5,000 reais	46	48.4	22	25.0	**0.003**
5,000-10,000 reais	23	24.2	37	42.0	
> 10,000 reais	26	27.4	29	33.0	
**Place of birth**^† **^					
< 200,000 inhabitants	49	51	36	39.6	0.115
> 200,000 inhabitants	47	49	55	60.4	
**Skin color**^‡ **^					
White	81	84.4	88	96.7	**0.004**
Black/brown	15	15.6	3	3.3	
**Child(ren)**^§^					
No	96	99.0	91	98.9	0.738
Yes	1	1.0	1	1.1	
**How were you born?**					
Vaginal delivery	44	45.4	41	45.1	0.966
Cesarean section	53	54.6	50	54.9	

^*^ Values obtained using the chi-square test and Fisher’s exact test, as appropriate.

^†^ Municipalities classified according to demographic data obtained from IBGE. [26].

^‡^ None of the students described themselves as being a native Indian.

^§^ Two abortions were reported by students in the sixth year.

** The total number of answers does not reach 100% because some of the students declined to answer some of the questions.

Table 2 shows the students’ responses to each one of the four questions. As shown, a greater proportion of sixth-year students expressed their preference for a Cesarean section for the birth of their own child compared to first-year students, this difference being statistically significant.

**Table 2:** Preferred mode of delivery according to the year of the medical course and circumstance analyzed

	**First year**		**Sixth year**		p-value^*^
	**n**	**%**	**n**	**%**	
**In a normal pregnancy**					
Vaginal delivery	91	94.8	81	88.0	0.097
Cesarean section	5	5.2	11	12.0	
*Total*^†^	*96*		*92*		
**In a pregnancy under your care**					
Vaginal delivery	86	89.6	85	93.4	0.350
Cesarean section	10	10.4	6	6.6	
*Total*^†^	*96*		*91*		
**In the general population, as a healthcare manager**					
Vaginal delivery	89	95.7	89	98.9	0.194
Cesarean section	4	4.3	1	1.1	
*Total*^†^	*93*		*90*		
**In your own pregnancy or that of your partner**					
Vaginal delivery	79	83.2	53	58.9	**< 0.001**
Cesarean section	16	16.8	37	41.4	
*Total*^†^	*95*		*90*		

^*^ Values obtained using the chi-square test or Fisher’s exact test, as appropriate.

^†^ The total number of answers does not reach 100% because some of the students declined to answer some of the questions.

To investigate whether some of the study variables affected the sixth-year students’ preference for that form of childbirth in the case of their own pregnancy or that of their partner, multiple binary logistic regression was performed (Table 3). According to this analysis, the year of the course was the only variable that positively affected preference for a Cesarean section, although it is impossible to state whether the odds ratio is exactly the same as the relative risk [27]. This approach can be used to eliminate any possible effect of variables that were not homogenous to the two groups of students: monthly family income, skin color and gender (Table 1), thus attributing greater importance to the variable concerning the year of medical school in which the student was enrolled. The questions for which the difference between the first and final year students was smaller were not submitted to the same statistical analysis. 

**Table 3:** Preference for Cesarean section for oneself or for one’s partner according to selected variables

**Variable**	**n**	**%**	Unadjusted OR^*^	**95%CI**^**†**^	**Adjusted OR**^**‡**^	**95%CI**^**†**^	**p-value**
**Year of the course**							
First	16	16.8	1				
Sixth	37	41.1	3.45	1.74–6.82	**2.93**	**1.03–8.30**	**0.044**
**Monthly family income**							
< R$ 5,000	14	20.6	1				
R$5,000–10,000	19	31.7	1.79	0.80–3.98	1.26	0.53–3.02	0.6
> R$10,000	20	39.2	2.49	**1.10–5.61**	1.88	0.78–4.55	0.16
**Skin color**							
Black/brown	1	5.6	1				
White	51	30.9	7.60	0.98–58.7	4.16	0.51–34.1	0.184
**Gender**							
Male	22	22.2	1				
Female	31	36.0	1.97	**1.03–3.77**	1.52	0.75–3.07	0.246
**Age group**							
≤ 23 years	19	29.4	1				
> 23 years	34	37.0	2.28	**1.18–4.41**	1.07	0.39–2.90	0.895

^*^Odds ratio by univariate logistic regression.

^†^ Confidence interval.

^‡^*Odds ratio* by multiple binary logistic regression.

The student’s preferred form of childbirth in the case of their own pregnancy or that of their partner was compared with their options in the different scenarios suggested. It was found that, when the student preferred a vaginal delivery for him/herself, he/she invariably chose the same mode of delivery in the other scenarios. However, when the student’s personal choice was for a Cesarean section, the responses given for the other scenarios differed in the majority of cases. Even if he/she preferred a Cesarean section in their own case, the majority of these students (69.2%) believed that normal childbirth offered fewer risks and conferred greater benefits; therefore, 70.6% would choose a vaginal delivery for a patient under their care and 89.8% considered vaginal childbirth better for the general population (data not shown in tables).

Analysis of the open question on why the student chose that particular form of childbirth showed that, when the choice was for a vaginal delivery, the reasons given were: “fewer risks for the mother and fetus (less morbidity and mortality)”, “natural/physiological/less aggressive” and “much faster recovery/with less pain”, both in the case of the first-year and sixth-year students. In addition, six students in the final year of the course emphasized the need for pain relief when choosing a vaginal delivery for the birth of their own children. Pain relief was not mentioned by any of the first-year students.

When Cesarean section was chosen for the birth of the student’s own child, the most common reason given was “less pain and/or less suffering”. Another reason given was the possibility of being able to plan the moment of childbirth, and this was mentioned both by first-year and by sixth-year students. Nine students in the sixth year and only one in the first year mentioned a fear of anatomical alterations (development of either anal or urinary incontinence, genital prolapse and lacerations). Three students (two in the sixth year and one in the first year) preferred a Cesarean section because this form of childbirth offered “fewer risks to the mother and fetus”.

## Discussion

Around 30% of the medical students who participated in this study stated that they would prefer a Cesarean section for the birth of their own child, with a significantly greater proportion of sixth-year students opting for this mode of delivery compared to the first-year students. A search of the Medline, SCIELO and LILACS databases conducted on October 31, 2011 failed to find any similar studies involving undergraduate medical students when the following keywords were used in English, Portuguese and Spanish: Cesarean section; medical education; delivery.

Although the difference in age between the two groups was predictable, it may constitute a confounding factor, since there was a mean difference of 4 years in age between the groups. It should be taken into consideration that the sixth-year students must have more experience and greater maturity with regard to their sexuality and may be closer to planning their own pregnancies. These differences between the groups may have affected the students’ answers irrespective of the effect of their medical training. Although no other studies on this subject (the form of childbirth preferred by medical students) are available in the literature, a web survey was applied to over 3,600 university students on their preference for type of childbirth. No comparison was made between age-groups; however, slightly less than 9% indicated a preference for a Cesarean section [28]. This percentage is similar to that found in the present study and to data presented in a meta-analysis, which show that in a population of more than 600 nulliparous, only 10.2% would opt for a Cesarean section [29].

In another study, 40 primigravidas of 16 to 30 years of age were interviewed prior to delivery. A desire for a Cesarean section was found in the same 10% of interviews; nevertheless all referred to women less than 21 years of age. It was also found that the preference for a Cesarean section increased from 8% in women with only primary school education to 11% in those with high-school education [30]. Taking age into consideration, the present study revealed an inverse trend, with an increase in the intention of having a Cesarean section at older ages. This same intention appeared to increase with the number of years of schooling in both studies; however, in the present study, the increase was much more significant. When these two aspects, age and schooling, are analyzed together, the hypothesis is strengthened that the preference for a Cesarean section is affected by learning up to the sixth year of medical school.

Comparison between the two groups (1^st^ and 6^th^ years) also revealed statistically significant differences with respect to skin color, gender and family income. The greater number of black or brown-skinned students and/or students with a lower family income among the first year students compared to the sixth-year students was already expected. In fact, this was a consequence of the affirmative action program implemented at UFSC in 2008 [31] in which 30% of university enrollment is reserved for candidates who attended public elementary and high schools, with a third of the enrollment being reserved for black candidates. The predominance of female students in the sixth year was unexpected and was considered to be the result of chance.

Overall, 41.4% of the sixth-year students reported that they would choose a Cesarean section for the birth of their own child. If we consider that the opinions expressed by the sixth year students are the same as those they would express as physicians following graduation, then the results found in the present study may be compared with data from studies on the personal preference of obstetricians with respect to childbirth. In contrast with the data obtained in the present study, the percentages of obstetricians in those studies who were in favor of the abdominal route of delivery for themselves or for their partner were in general much lower. The reported proportions were 1.1% in Denmark, [32] 1.4% in the Netherlands, [33] 2% in Belgium, [34] 7% in Ireland, [35] 11.3% in Australia/New Zealand [36] and 15% in the United Kingdom. [37] Only one study reported a preference rate for Cesarean section of over 40%. In a study carried out in districts of the United States, Gabbe and Holzman [38] reported that 46.2% of the obstetricians interviewed were in favor of a Cesarean section for their own pregnancy even if there was no medical indication for this procedure. To the best of our knowledge, no such studies have been conducted in Brazil.

Considering that medical internship is one of the most significant segments of medical training, it is logical to imagine that physicians graduating from medical school who have undergone specialist training in obstetrics and gynecology in maternity hospitals in which Cesarean section rates are high would be more liberal in their opinions and conduct in relation to this mode of delivery. It has been suggested that the way in which medical students experience labor (in theoretical classes but mainly while undergoing training in obstetric centers in which Cesarean section rates are higher than recommended) would lead the future professional to fear vaginal delivery. The reason for this uneasiness would lie both in a lack of experience with the normal progression of labor, the suffering attributed to vaginal delivery and in a lack of knowledge on the possible complications of a Cesarean section. Coincidentally or not, the Cesarean section rates presented by the maternity hospitals in which the UFSC medical students undergo obligatory training are similar to the percentage of sixth-year students who reported preferring a Cesarean section for their own pregnancy.

In 1996, Rattner [39] reported an increasing number of abdominal deliveries in university teaching hospitals in the state of São Paulo and reinforced the relevance of exposing students to the progression of normal childbirth in these establishments. Moraes and Goldenberg were also concerned with the effect of medical training on Cesarean section rates [40]. Analyzing the discourse of those involved in the education process at the São José do Rio Preto School of Medicine, these investigators highlighted the value given by the faculty to Cesarean sections: “*for the physician it is easier and quicker to perform a Cesarean section. Furthermore, the procedure offers no greater risk than those associated with a normal delivery*”. Moreover, the study in question concluded that undergraduate students and residents, despite formally acknowledging the merits of the vaginal route of delivery, proved to be convinced that in practice it was not feasible: “*our professors teach us that Cesarean sections should only be performed as a last resort*”; nevertheless, “*after we graduate, it’s a whole different scenario*”. Data presented by Burns, Geller and Wholey [41] in 1995, based on a study that evaluated the physician’s role in the decision to perform a Cesarean delivery, ratified the relevance of the attitude of teaching hospitals, showing that physicians appear to bear the imprint of the institution in which they underwent their technical training in their future conduct.

Comparing the respondent’s preferred mode of delivery in the case of her own pregnancy (or that of a partner in the case of male respondents) with his/her preference in other scenarios, a difference was found between those who preferred a Cesarean section for themselves and those who preferred vaginal delivery. Hantoushzadeh et al. [42] conducted a study in Iran with 785 female obstetricians and reported that 28.3% of the professionals who stated having recommended vaginal delivery for their patients would choose a Cesarean section for themselves. In the present study, 69.2% of those who reported that they would choose a Cesarean section for their own delivery also stated that in their opinion vaginal delivery involves fewer risks and confers greater benefits in a normal pregnancy.

Moraes and Goldenberg [40] speculated that, although students are able to discourse on the advantages of vaginal delivery (perhaps learned from their theoretical classes), when they are asked to make a personal choice, many appear to think again, possibly as a consequence of their practical experience in obstetrics during their university training. On the other hand, this type of contradiction was not found among the students who reported a preference for a vaginal delivery in the case of their own pregnancy.

Although the majority of the students who would choose a Cesarean delivery for themselves or for their partner would recommend vaginal delivery for their patients when no pathologies are present, a personal choice for a Cesarean section was found to result in a trend of the individual choosing that same mode of delivery for his/her patients (p < 0.001). This finding is reinforced by the observation of McGurgan, Coulter-Smith and O’Donovan, [35] who reported a strong association between a personal preference of obstetricians for Cesarean section and the Cesarean section rates in the hospitals in which they worked. Koken et al. [20] raised the hypothesis that healthcare professionals who prefer Cesarean sections for their own pregnancies are less tolerant of the risks that vaginal delivery represents for the fetus. In addition, Hopkins [11] conducted a qualitative study in Brazil and reported that many physicians in the country, despite outwardly defending vaginal delivery, do not usually wait for delivery to progress naturally and anticipate the line of action even when no relevant clinical indications are present. Hence, Cesarean sections are indicated for convenience or esthetical reasons and also due to a fear of vaginal delivery, neither of which is based on the best available evidence. The above-mentioned findings suggest that this may be the future conduct of these respondents and that this attitude may at least be partially responsible for the rate of Cesarean sections in Brazil.

Because of its quantitative design, the present study was unable to evaluate the motivations behind the choice of one mode of delivery or the other in greater detail. Analysis of the reasons given by the groups, however, provides some insight into this question. The importance given to the pain and suffering attributed to vaginal delivery is noteworthy. Institutional violence against women occurs in maternity hospitals [43] and the students may have witnessed this during their practical training, confusing this suffering imposed by the system with the inherent pain of childbirth. A fear of anatomical changes was also found, reported principally by sixth-year students. The high percentage of episiotomies performed during vaginal deliveries, together with the high rate of Cesarean sections in the country may play a role in shaping the view of these professionals. [44] In the majority of studies published in the literature on the personal preference of obstetricians with respect to the mode of delivery they would wish for themselves, fear of perineal damage figured as the principal justification for having chosen an elective Cesarean section [19,32,34,36,37,44,45]. Only three publications were found that mentioned pain as a reason for choosing a Cesarean section. [34,36,37] The question of being able to schedule the time of giving birth was also mentioned. This aspect has not been evaluated in any depth; however, it appears to play an undeniably important role in the high rates of Cesarean section in supplementary healthcare in Brazil [17,37].

Some limitations of this study include the cross-sectional design that does not allow causal relationships to be determined, and the approach used to eliminate any possible effect of variables that were not homogenous to the two groups of students: monthly family income, skin color and gender, thus attributing greater importance to the variable concerning the year of medical school in which the student was enrolled.

The present study provides some insight into the probable effect of medical education on the increasing rates of Cesarean sections. In brief, the students are probably being socialized into a medicalized birth culture during training. It should be emphasized that the questions proposed compared a Cesarean section in a woman with no pathologies to a vaginal delivery without providing further details with respect to the procedures (e.g. the setting in which the vaginal delivery would occur). Although it is impossible to extrapolate these findings to all the medical schools in the country, the similarities in the structure of the curriculum, particularly in public institutions, should be emphasized. Further studies should be conducted with students in other courses in health-related and other areas and with individuals of the same age bracket who are not university students in order to provide greater depth to this discussion. This should motivate multicenter studies, particularly longitudinal and qualitative studies, which would be of great value in characterizing the moment at which final year students adopt such a permissive attitude in relation to Cesarean sections as an option for childbirth and their reasons for doing so.

In view of the need for alternatives to reduce one of the highest Cesarean section rates in the world, a study conducted to identify possible causes is of the greatest relevance. Obstetric care in teaching hospitals in Brazil may require changes in order to provide the medical student with a clearer view of the benefits of vaginal childbirth for the mother and the fetus, as well as structured prenatal psychological preparation of the pregnant woman for the moment of birth and acceptance of delivery as a natural, physiological event. The humanization of care at childbirth and a positive experience of this moment may help change this scenario.

## Conclusions

The majority of medical students would recommend vaginal delivery because this form of childbirth offers fewer risks and confers greater benefits in a low risk pregnancy. This would be their recommendation as trained physicians caring for women with a normal pregnancy and also what they would recommend, as a healthcare manager, for the general population. There was no statistically significant difference between the options made by the students in the first and final year of the medical course with respect to these questions.

The majority of students would also choose vaginal delivery for the birth of their own child. In this case, however, a greater proportion of the sixth-year students compared to the first-year students preferred a Cesarean section. This indicates a probable effect of medical education on a permissive culture of Cesarean sections as an option for childbirth.

## Competing interests

The authors declare that they have no competing interests.

## Authors’ contributions

TW, RK and GS designed the study. TW and RK developed the questionnaire. TW and GS carried out data collection. TW, RK and EO performed the statistical analysis. All authors interpreted the results, drafted the manuscript and read and approved the final manuscript.

## Pre-publication history

The pre-publication history for this paper can be accessed here:

http://www.biomedcentral.com/1472-6920/12/57/prepub
